# Non-steroidal Anti-inflammatory Drugs and Osteopathic Manipulative Treatment for Pain Management in Patients With Osteoarthritis: A Literature Review

**DOI:** 10.7759/cureus.44168

**Published:** 2023-08-26

**Authors:** Veenah Stoll, Jennifer M Jost, Allyson Jack, Timothy Johnson, Sarah Klein, Jake Darbhanga, Adam Hurwitz, Rohit S Mehra, Holly B Waters

**Affiliations:** 1 Dr. Kiran C. Patel College of Osteopathic Medicine, Nova Southeastern University, Clearwater, USA; 2 Dr. Kiran C. Patel College of Osteopathic Medicine, Nova Southeastern University, Fort Lauderdale, USA

**Keywords:** topical nsaid, osteopathic manipulative treatment, pain management, nsaids, chronic pain, omt, diclofenac, joint pain, omm, osteoarthritis

## Abstract

The pathophysiology of osteoarthritis (OA) involves the destruction of articular cartilage and the overgrowth of bone with lipping and spur formation. Nerve endings in the joint capsule and adjacent tissues play a major role in the pain mechanisms of osteoarthritis. This often requires patients to seek pain control measures beyond over-the-counter drugs, such as local anesthetics. Osteopathic manipulation treatment (OMT) is a conservative, non-pharmacological treatment that can be used to help treat chronic pain associated with OA. Other non-pharmacologic therapies include weight loss, exercise, physical therapy (PT), and assistive devices. However, pharmacologic management may be added synergistically to control flares and maintain baseline activities of daily living. While oral non-steroidal anti-inflammatory drugs (NSAIDs) have been the mainstay of treatment for pain and inflammation associated with OA, they have a non-selective inhibitory action that often results in negative side effects when used chronically. The possibility of minimizing these complications through alternate treatments such as topical NSAIDs provides an opportunity for patients to receive adequate pain relief from OA without suffering unnecessary consequences. This literature review seeks to assess the state of research regarding topical NSAIDs and OMT as alternatives to the current gold-standard treatment of OA. The significant inclusion criteria consisted of articles that described the effects of OMT on OA or the use of topical NSAIDs such as Voltaren on OA. Due to the limited articles found, a qualitative analysis was performed, and the salient conclusions are outlined. Alternative pharmacological and non-pharmacological treatments, such as topical diclofenac gel and OMT, have shown promising results in the treatment of pain in OA. It is seen that a majority of patients achieve pain management using NSAIDs, acetaminophen, or topical analgesics. Both diclofenac sodium and OMT have individually been shown to be effective treatments of OA when compared to the use of oral NSAIDs. A holistic treatment approach that utilizes both topical diclofenac sodium and OMT may provide OA patients with an effective option to reduce their moderate to severe chronic pain with limited side effects. Further, high-quality randomized controlled trials are needed to identify whether synergistic effects occur when combining diclofenac sodium gel and OMT for pain relief in patients with OA.

## Introduction and background

Osteoarthritis (OA) is the most common joint disorder in the United States, with an estimated 37% and 27% of people over the age of 60 having hand and knee joints affected, respectively [1]. The pathophysiology of OA involves the destruction of articular cartilage and the overgrowth of bone with lipping and spur formation. These changes often lead to inflammation, chronic pain, and impaired function in daily life [2]. The chronic pain seen in OA can originate from several different locations, including, but not limited to, periarticular muscles and ligaments, synovial membranes, joint capsules, periosteum, and subchondral bone [3]. Nerve endings in the joint capsule and adjacent tissues play a major role in the pain mechanisms of OA [4]. This often requires patients to seek pain control measures beyond over-the-counter drugs, such as local anesthetics. Other factors contributing to the discomfort found in OA include the growth of osteophytes, stretching of the local periosteum, microfractures, synovitis, and increased intraosseous pressure [4].

Clinical goals for OA involve reducing pain and inflammation, minimizing impact on daily life, and halting the progression of joint deterioration. Non-pharmacological or conservative treatment is the recommended first-line approach to therapy. Osteopathic manipulation treatment (OMT) is one of the conservative, non-pharmacological treatments that can be used to help treat chronic pain associated with OA. OMT is performed by a Doctor of Osteopathic Medicine (D.O.), targeting fascia, musculature, joints, nerves, and surrounding tissue that can ultimately provide pain relief and improved functionality [5]. A complete osteopathic structural exam should be performed and may include monitoring the patient’s gait, assessing bony and muscular landmarks, and assessing for tissue texture changes [3]. The physical manipulation used in OMT allows physicians to address anatomical and physiological pathology. Other non-pharmacologic therapies include weight loss, exercise, physical therapy (PT), and assistive devices [4,5]. However, pharmacologic management may be added synergistically to control flares and maintain baseline activities of daily living [5].

While oral non-steroidal anti-inflammatory drugs (NSAIDs) have been the mainstay of treatment for pain and inflammation associated with OA, NSAIDs have a non-selective inhibitory action that often results in negative side effects when used chronically [5]. These may include gastritis, peptic ulcers, kidney disease, heart failure, meningitis, and cognitive dysfunction [6]. More than 100,000 patients are hospitalized each year due to gastrointestinal complications related to NSAID use, and approximately 16,500 of these patients die annually from subsequent complications [7]. Although NSAID use is very common with chronic diseases such as OA, these aforementioned side effects are often more severe in the elderly population due to their reduced metabolic activity and lower physiologic reserve [6]. The possibility of minimizing these complications through alternate treatments, such as topical NSAIDs, provides an opportunity for patients to receive adequate pain relief for their OA without suffering unnecessary consequences. This literature review seeks to assess topical NSAIDs and OMT as alternatives to the current gold standard treatment of OA.

This research was previously published as an abstract in the Southern Medical Journal in July 2022. This research was also a poster presented at the Southern Medical Association in February 2022.

## Review

Methods

The following databases were searched on August 18, 2021: EMBASE, EBSCO databases (CINAHL, SPORTDiscus, Cochrane, Global Health, Ageline), PubMed, Web of Science, and Google Scholar. Search terms included (("non steroidal anti inflammatory" OR "nonsteroidal anti inflammatory" OR "nonsteroidal antiinflammatory" OR “non steroidal anti inflammatory” OR "nsaid" OR “nsaids” OR "feloran" OR "voltarol" OR "ortofen" OR "orthophen" OR "orthofen" OR "diclofenac" OR "voltaren") AND (osteopath*)). The significant inclusion criteria consisted of articles that described the effects of OMT on OA or the use of topical NSAIDs such as Voltaren or OA. 

The search yielded 443 citations. After deleting duplicates, 436 articles remained. Titles and abstracts were reviewed for the use of topical NSAIDs or OMT in OA treatment or the treatment of chronic pain. Three reviewers were blinded in decisions of title and abstract sorting, and the majority rule was used to include or exclude articles that did not have a unanimous decision. The title and abstract review excluded an additional 371 articles. Full-text review of 45 articles resulted in 16 articles being utilized for the final review. Of these 16 articles reviewed, only four explicitly mention the use of OMT in the management of OA, and only one of those contained original research. All articles, including all published randomized clinical trials (RCT) and reviews of the literature that mentioned OMT or topical NSAIDs, were included in the final review. Five additional articles were added through reference skimming, and three additional articles were selected through direct searching for a total of 24 articles. Due to the limited articles found, a qualitative analysis was performed, and salient conclusions were outlined. This inclusion process can be illustrated in Table 1 as below.

**Table 1:** Inclusion and Exclusion Criteria for the Articles Reviewed

	Inclusion Criteria	Exclusion Criteria
Population	Articles for patients with a diagnosis of OA of the following joint: ankle, knee, foot, hand, wrist, or elbow. Patients with chronic pain.	Patients <18 years old, patients with a diagnosis of active cancer, acute strain or injury, rheumatoid arthritis or psoriatic arthritis diagnosis, low back or neck pain diagnosis, patients currently pregnant, post-infectious sequelae, patients diagnosed with COVID or other active infections, patients treated with injectable/intramuscular medications or other treatments that did not include topical NSAIDs or OMT.
Concept	Articles about topical NSAID treatments for OA Articles about OMT treatment results for OA	Books
Context	Common practices in the outpatient setting	Hospice/palliative care, other pathologic causes for impaired mobility

Results

Non-pharmacological Management

Management of osteoarthritic pain involves a diverse range of treatment options, tailored to each patient. Patients with chronic pain may respond more successfully to a multifaceted approach than to a single treatment method. For example, the addition of non-pharmacologic therapies used in conjunction with medication ensures that multiple aspects of chronic pain are being addressed through treatment [8]. In the past, pharmacotherapy of OA has been supplemented with a multitude of non-pharmacological treatment modalities, including patient education, PT, OMT, and weight loss [9]. Combinations of nonpharmacologic management, such as spinal manipulation, OMT, and PT, have been shown to decrease pain and improve function in patients suffering from chronic knee pain [3,8].

There are four main categories OMT techniques are divided into. These include direct, indirect, active, and passive techniques. Direct techniques engage the barrier of the somatic dysfunctions, while indirect techniques involve placing the patient in a position of ease. Active and passive treatments refer to the patient assisting in the treatment or relaxing while the provider moves their body, respectively [10]. OMT techniques that have been used for AO of the knee include muscle energy technique (MET), counterstrain (CS), facilitated positional release (FPR), high-velocity low amplitude (HVLA) techniques, and myofascial release (MFR) [3]. 

MET includes two main modalities: post-isometric relaxation and reciprocal inhibition. Post-isometric relaxation is a direct and active technique. It is theorized that stretching the Golgi tendon organ allows the relaxation of the agonist's muscle. This encourages further movement into the patient’s barrier for a greater range of motion of a joint or set of musculature [10]. Reciprocal inhibition is another active technique that can be direct or indirect that engages the antagonist muscle to relax the somatic dysfunction of the agonist muscle [10]. MET can be used for a wide variety of dysfunctions caused by OA, as seen in the OMT management of post-knee replacement surgery [11]. MET can be used in nearly any joint affected by OA, especially dysfunctions of the tibiofemoral joint, knee extension, knee flexion, and internal or external rotation [3].

Counterstrain is an indirect and passive technique in which the provider identifies a tender point. Treatment is then initiated by shortening the muscle involved in the somatic dysfunction to achieve muscle relaxation and pain reduction of the tender point. Although indirect, counterstrain can have similar applications to MET. Shown to be effective in the management of post-knee replacement surgery, counterstrain can also be used to treat OA-induced tender points in the medial and lateral knee for pain reduction [3,11].

FPR is an indirect and passive technique in which the provider passively moves the patient into their position of ease, applies an activating force, and then passively moves the patient further into their position of ease [10]. FPR can be used to treat superficial muscles and deep, intervertebral muscles that influence vertebral motion [10]. FPR has been shown to treat numerous tender points caused by OA along the tibiofemoral joint [3].

HVLA is a direct and passive technique in which the patient is passively moved into their restrictive barrier and then a high velocity, low amplitude thrust is applied through the barrier, similar to chiropractic spinal manipulation [10]. HVLA forces are typically applied to realign bones and joints. There are two theories behind HVLA: first, that the thrust forcefully stretches a contracted muscle, producing a load of afferent impulses from the muscle to the central nervous system, reflexively sending inhibitory impulses causing muscle relaxation; second, that the thrust forcefully stretches the contracted muscle, activating Golgi tendon receptors to relax the muscle [10]. HVLA is another versatile technique used in the management of post-knee replacement surgery, as well as several somatic dysfunctions manifesting from OA at the fibular head or lateral knee [3,11].

MFR can be indirect or direct and passive or active. Using three planes of motion (traction, rotation, and compression), the patient’s tissues are placed in either a position of restriction or ease. This will activate or relax the dysfunctional tissues, respectively [10]. In general, MFR techniques help to restore motion, relieve edema and pain, improve circulation and lymphatic flow, and support visceral function [10]. With both direct and indirect applications, MFR has the ability to treat a wide variety of patients and diseases. MFR has been shown to assist in the healing process of many post-operative conditions, including knee replacement surgery, patellofemoral pain syndrome, and OA [3,11].

Due to the multifaceted effects of OMT, many of these interventions have a wide variety of applications. Providers are able to address edema, pain, circulation, lymphatic flow, range of motion, muscular somatic dysfunctions, and other restrictions in the knee joint that may be contributing to pain and poor function in patients with OA. Additionally, many OA patients may benefit from the multisystem effects of OMT. Using manipulation to treat the joints both above and below the knee, including the lumbar spine, ankle, and pelvis, significantly improved functional outcomes of OA and delayed the need for surgery in applicable patients [12]. When compared to placebo groups, after eight weeks of treatment, there was both a clinically and statistically significant improvement in the six-minute walk distances by 13.1%. Western Ontario and McMaster Universities Osteoarthritis Index (WOMAC) scores also improved by 55.8% compared to baseline (p<0.05) [12].

As indicated by our initial search, there is a scarcity of studies that analyze and provide evidence for the direct effectiveness of OMT in the treatment of OA (Table 2). In this case, we can analyze OMT in the treatment of other disorders of the knee, such as total knee replacement recovery or patellofemoral pain syndrome. It may be applicable to apply the techniques and concepts from these chronic pain pathologies to the treatment of OA. A review that studied the effectiveness of OMT in total knee arthroplasty recovery following severe OA found manual lymphatic drainage with effleurage or lymphatic pump techniques helped control edema [11]. Effleurage and lymphatic pump techniques are indirect and passive modalities that improve lymphatic drainage through gentle or rhythmic pressure, respectively. The investigators argue that edema can mimic ischemia by creating a barrier that restricts blood flow due to the accumulation of inflammatory fluid. This optimized lymphatic drainage and blood flow then improves healing and reduces pain in the dysfunctional joint [11]. Table 2 presents the non-pharmacologic treatment of chronic knee pain syndromes.

**Table 2:** Non-pharmacologic Treatment of Chronic Knee Pain Syndromes

Author	Population	Design	Results	Conclusion
Zhou et al. [11]	18 studies	This was a systematic review of the literature. A search was conducted of the National Library of Medicine's MEDLINE/PubMed databases in addition to the Journal of American Osteopathic Association with keywords “post-operative”, “knee arthroplasty”, “total knee replacement”, and “osteopathic medicine”.	A total of 18 studies were found, including 10 prospective studies, 3 case reports, 2 cross-sectional studies, 1 case-control study, and 2 reviews of the literature.	OMT can provide potential therapeutic benefits following total knee arthroplasty and can provide pain relief, increased range of motion, and improved function in daily living.
Deyle et al. [12]	83 patients with knee OA	The treatment group received manual therapy of the knee, lumbar spine, hip, and ankle. The placebo group received ultrasound therapy. Both groups were treated twice weekly for four weeks.	Clinically and statistically significant improvements in 6-minute walk distance and WOMAC score at 4 weeks and 8 weeks. At one year, only 5% of patients in the treatment group underwent total knee arthroplasty, while 20% of the placebo group did.	A combination of manual therapy and exercise had functional benefits in OA of the knee and may delay the need for surgery.
Tramontano et al. [13]	35 patients with patellofemoral pain syndrome.	Randomized, controlled, and single-blinded trial with four sessions of OMT or four sessions of manual placebo intervention that consisted of passive touching without joint mobilization in a protocolled order. Focus was put on 10 anatomical regions and treatment of the somatic dysfunctions found.	There were significant differences in the VAS scores in those who received treatment.	OMT can lead to pain reduction in patients with patellofemoral pain syndrome.

A study in patients with patellofemoral pain syndrome used articular and myofascial techniques, balanced ligamentous tension, visceral manipulations, and osteopathy in the cranial field to treat full-body somatic dysfunctions. They found that there was a significant reduction in pain between the OMT and placebo groups [13]. These investigators concluded that a full-body treatment approach to single joint dysfunctions can better alleviate pain. Their results indicate that the mechanisms initiated and maintained by the primary dysfunctional area. For example, a sacral dysfunction may induce pain in the knee [13].

Pharmacological Management

Oral NSAIDs are commonly recommended for the relief of OA symptoms because of their effectiveness, anti-inflammatory effect, and lesser addictive potential when compared to opioids [14]. The specific NSAID prescribed is usually selected based on pain intensity, inflammation, and gastrointestinal and cardiovascular risks of the patient [15]. NSAIDs non-selectively inhibit cyclooxygenase (COX) synthesis in the peripheral and central nervous systems. COX is the rate-limiting enzyme in the conversion of arachidonic acid to prostaglandin H2, the precursor to prostaglandins, prostacyclin, and thromboxanes, which are involved in the inflammatory response. COX-1 promotes homeostasis in the gut, kidney, and endothelium, while COX-2 is an inducible enzyme active in the inflammatory process. COX-2-specific NSAIDs maximize the anti-inflammatory effects while minimizing dysregulation of the gut, kidney, and epithelium [16].

Alternative pharmacologic options to oral NSAIDs may include a topical NSAID, such as diclofenac gel. Diclofenac gel has been shown to be well-tolerated while also providing relief against osteoarthritic knee pain [8,17]. In a systematic review of four randomized controlled trials, topical diclofenac sodium was associated with a statistically significant improvement in WOMAC pain (SMD was -0.33 (95% CI -0.48 to -0.18)), stiffness (SMD of -0.30 (95% CI -0.45 to -0.15)), physical functionality (SMD of -0.35 (95% CI -0.50 to -0.20)), and patient global assessment (SMD was -0.39 (95% CI -0.54 to -0.24)) [17]. The localized action of topical NSAIDs avoids the stomach and first-pass effects in the liver, providing a more favorable side effect profile than their oral counterparts [18]. Studies regarding the use of topical diclofenac showed greater effectiveness than placebo with similar results to oral diclofenac [19-21]. In a 12-week trial, diclofenac sodium gel was associated with significant improvements in WOMAC pain scores, WOMAC physical function, global rating of disease, and pain on movement in a modified efficacy subpopulation group [20]. In another randomized, double-blind, vehicle-controlled 12-week trial, diclofenac sodium gel showed a statistically significant reduction in WOMAC pain (p=0.01), WOMAC physical function (p=0.001), and mean global rating of disease (p<0.001) starting at week 1 [21]. Furthermore, an additional double-blind, placebo-controlled three-week trial showed that diclofenac was significantly more effective in treating pain in OA of the knee (p=0.03) [22].

Other topical medications, such as the root and stem bark derived from the Daphne giraldii Nitsche, have also shown benefits for OA patients. Cortex Daphnes, or Zushima patches, are an alternative treatment being explored that has been shown to provide similar effects to topical NSAIDs when used for OA of the knee [23]. While not every topical drug was shown to be as effective as diclofenac gel, various studies that investigated the effectiveness of topical medications for OA patients all noted fewer systemic side effects when compared to oral medication [24]. Unfortunately, little research has been done on the effectiveness of topical NSAIDs for OA patients in the United States compared to that in other countries. Furthermore, diclofenac topical gel is the only topical NSAID currently approved by the Food and Drug Administration. The promising results of topical diclofenac indicate more research is needed comparing its effectiveness to alternatives in the field. Table 3 presents the pharmacological management of chronic knee syndromes.

**Table 3:** Pharmacological Management of Chronic Knee Syndromes

Author	Population	Design	Results	Conclusion
Towheed [13]	4 articles	Systematic review of the literature	In comparison to a vehicle control placebo (VCP), there were statistical significant improvements for the WOMAC pain, stiffness, and physical function subscales, as well as for patient global assessment in Pennsaid use.	Pennsaid is an effective topical NSAID in OA of the knee.
Klinge et al. [18]	9 studies including 2403 patients studied topical vs. oral NSAIDs	This is a comprehensive review of topical and oral NSAIDs that analyzed comparative clinical trials and meta-analyses.	Topical and oral NSAIDs displayed similar efficacy. Oral NSAIDs had more GI side effects.	Topical NSAIDs are a comparable alternative to oral NSAIDs and have fewer adverse effects.
Baraf et al. [20]	420 patients were assigned to vehicle or topical diclofenac sodium gel	208 patients were assigned to topical diclofenac sodium gel and 212 patients were assigned to the vehicle group. 4 g of the assigned treatment was applied to the knee 4 times daily.	Topical diclofenac sodium provided a statistically significant reduction in WOMAC pain and physical function.	Topical diclofenac sodium provided pain relief and physical function improvement in OA of the knee.
Barthel et al. [21]	492 adults with knee OA	Randomized, double-blind, vehicle-controlled trial in which 254 patients applied topical diclofenac and 238 patients applied a vehicle 4 times daily for 12 weeks.	Topical diclofenac had a significant decrease compared to the vehicle group in WOMAC pain, physical function, and mean global rating of disease.	Topical diclofenac is an effective treatment for OA of the knee.
Niethard et al. [22]	238 patients with knee OA	This was a randomized, double-blind, placebo-controlled, multicenter study of 3-week duration in which 117 patients applied 4 g of topical diclofenac and 121 patients applied placebo gel.	Scores for all 3 WOMAC ratings were statistically superior to placebo.	Diclofenac gel is a safe and effective treatment modality for knee OA.
Li [23]	264 patients with symptomatic knee pain	This was a multicenter, randomized, parallel-group study comparing the Cortex Daphnes patch with topical nonsteroidal anti-inflammatory drugs in patients with knee OA. Cortex Daphnes or indomethacin cataplasms were applied to the knee once daily for 2 weeks.	Cortex Daphnes was non-inferior to the topical NSAID. No significant changes were found between groups except WOMAC pain was higher in the Cortex Daphnes group.	Cortex Daphnes provides enhanced physical function and improved quality of life with some pain relief in those with knee OA.

Due to financial feasibility, only articles that were freely accessible to the public or available through the Nova Southeastern University Library were included. Additionally, the criteria excluded a variety of comorbidities and acute events to minimize conflicting data. This limits the generalizability and application of our results to the general population as most OA patients are older with multiple comorbidities. Limitations in the studies reviewed included, but are not limited to, small sample sizes, lack of comparison to groups receiving pharmacologic treatment, no explicit mention of OMT as a treatment modality, patient use of analgesics and muscle relaxants on an as-needed basis, and a lack of OMT placebo treatment. Therapeutic touch has known benefits and is typically used as a placebo treatment for OMT.

## Conclusions

Alternative pharmacological and nonpharmacological treatments, such as topical diclofenac gel and OMT, have shown promising results in the treatment of pain in OA. Studies show that a majority of patients achieve adequate pain management using NSAIDs, acetaminophen, or topical analgesics. This may be due to pharmacologic treatments of OA having been more thoroughly studied, recommended, readily available, and easy to use. In contrast, there is a paucity of research studying nonpharmacologic treatments for OA, making it difficult to compare the effectiveness of this treatment to the more well-established pharmacologic modalities. While clinical trials studying the knee joint have shown improvement in function and pain reduction after incorporating OMT into their treatment plan, there is still a gap in research regarding the effectiveness of OMT in treating other joints affected by OA.

Due to the nature of the study and limited current research supporting the use of OMT, no definitive conclusions can be drawn; however, OMT is an underutilized treatment modality. While the use of OMT in the treatment of OA has been very sparsely addressed in the literature, a plethora of studies have demonstrated the variety of applications in which OMT can decrease pain and stimulate healing. Therefore, general principles utilized in OMT for the treatment of a wide variety of pathology can be extrapolated to OA. Ultimately, we postulate that a holistic, individualized combination of pharmacologic treatment, such as topical diclofenac sodium gel, and OMT may be an effective option in the reduction of moderate to severe chronic pain caused by OA with limited side effects. It stands to reason that the improved biomechanics and decreased inflammatory load placed on the musculoskeletal system provided by OMT would be magnified when used in conjunction with a topical NSAID. The elderly population would particularly benefit from the proposed treatment combination of topical NSAIDs and OMT since they suffer from a disproportionately high incidence of both polypharmacy and contraindications to oral NSAIDs. Further, high-quality randomized controlled trials and clinical trials are needed to identify whether synergistic effects are observed with this treatment combination.

## References

[1] Zhang Y, Jordan JM (2010). Epidemiology of osteoarthritis. Clin Geriatr Med.

[2] College of Osteopathic Medicine (2019). COM Outlook Winter 2019. COM Outlook.

[3] Datta R, Burina L, Romanelli F, Flaum TB (2017). Knee pain in adults with an osteopathic component. Osteopath Fam Physician.

[4] Barron M, Rubin B (2007). Managing osteoarthritic knee pain. Journal of Osteopathic Medicine.

[5] Deveza LA (2018). Overview of the management of osteoarthritis. UpToDate.

[6] Marcum ZA, Hanlon JT (2010). Recognizing the risks of chronic nonsteroidal anti-inflammatory drug use in older adults. Ann Longterm Care.

[7] Fine M (2013). Quantifying the impact of NSAID-associated adverse events. Am J Manag Care.

[8] Rosenquist EW, Ellen E Overview of the treatment of chronic non-cancer pain. https://www.academia.edu/download/62517883/Overview_of_the_treatment_of_chronic_non-cancer_pain_-_UpToDate20200328-81409-1ejcdn0.pdf.

[9] (2021). Mayo Clinic: Osteoarthritis. Mayo Clinic Foundation.

[10] Savarese RG, Capobianco JD, Cox JJ (2003). Omt Review: [A Comprehensive Review in Osteopathic Medicine, 3rd ed]. Omt Review : A Comprehensive Review in Osteopathic Medicine. 3rd ed. Place of publication not identified: Savarese.

[11] Zhou Y, Chin J, Evangelista A, Podger B, Wan PJ, Lomiguen CM (2022). Inhibiting the musculoskeletal pathological processes in post-knee replacement surgery with osteopathic manipulative treatment: a systematic review. Cureus.

[12] Deyle GD, Henderson NE, Matekel RL, Ryder MG, Garber MB, Allison SC (2000). Effectiveness of manual physical therapy and exercise in osteoarthritis of the knee. A randomized, controlled trial. Ann Intern Med.

[13] Tramontano M, Pagnotta S, Lunghi C, Manzo C, Manzo F, Consolo S, Manzo V (2020). Assessment and management of somatic dysfunctions in patients with patellofemoral pain syndrome. J Am Osteopath Assoc.

[14] DeAngelo NA, Gordin V (2004). Treatment of patients with arthritis-related pain. J Am Osteopath Assoc.

[15] Fendrick AM, Greenberg BP (2009). A review of the benefits and risks of nonsteroidal anti-inflammatory drugs in the management of mild-to-moderate osteoarthritis. Osteopath Med Prim Care.

[16] Argoff Argoff, CE CE (2002). Pharmacologic management of chronic pain. J Am Osteopath Assoc.

[17] Towheed TE (2006). Pennsaid therapy for osteoarthritis of the knee: a systematic review and metaanalysis of randomized controlled trials. The. J Rheumatol.

[18] Klinge SA, Sawyer GA (2013). Effectiveness and safety of topical versus oral nonsteroidal anti-inflammatory drugs: a comprehensive review. Phys Sportsmed.

[19] Stanos S (2013). Osteoarthritis guidelines: a progressive role for topical NSAIDs. J Osteopath Med.

[20] Baraf HS, Gold MS, Clark MB, Altman RD (2010). Safety and efficacy of topical diclofenac sodium 1% gel in knee osteoarthritis: a randomized controlled trial. Phys Sportsmed.

[21] Barthel HR, Haselwood D, Longley S, Gold MS, Altman RD (2009). Randomized controlled trial of diclofenac sodium gel in knee osteoarthritis. Semin Arthritis Rheum.

[22] Niethard FU, Gold MS, Solomon GS, Liu JM, Unkauf M, Albrecht HH, Elkik F (2005). Efficacy of topical diclofenac diethylamine gel in osteoarthritis of the knee. Journal of Rheumatology.

[23] Li YT (2021). Clinical efficacy of cortex daphnes (Zushima) patch in patients with symptomatic knee osteoarthritis: a multicenter non-inferiority randomized controlled clinical trial. Front Pharmacol.

[24] Bolin DJ (2003). Transdermal approaches to pain in sports injury management. Curr Sports Med Rep.

